# The influence of sham feedback on physiological processing during fear-driven stimulation

**DOI:** 10.1371/journal.pone.0251211

**Published:** 2021-05-05

**Authors:** Jan Ehlers, Janine Grimmer, Veronika Strack, Anke Huckauf

**Affiliations:** 1 Department of Computer Science, Bauhaus-Universität Weimar, Weimar, Germany; 2 Department of General Psychology, Ulm University, Ulm, Germany; University of Toyama: Toyama Daigaku, JAPAN

## Abstract

Biofeedback constitutes a well-established, non-invasive method to voluntary interfere in emotional processing by means of cognitive strategies. However, treatment durations exhibit strong inter-individual variations and first successes can often be achieved only after a large number of sessions. Sham feedback constitutes a rather untapped approach by providing feedback that does not correspond to the participant’s actual state. The current study aims to gain insights into mechanisms of sham feedback processing in order to support new techniques in biofeedback therapy. We carried out two experiments and applied different types of sham feedback on skin conductance responses and pupil size changes during affective processing. Results indicate that standardized but context-sensitive sham signals based on skin conductance responses exert a stronger influence on emotional regulation compared to individual sham feedback from ongoing pupil dynamics. Also, sham feedback should forego unnatural signal behavior to avoid irritation and skepticism among participants. Altogether, a reasonable combination of stimulus features and sham feedback characteristics enables to considerably reduce the actual bodily responsiveness already within a single session.

## Introduction

Biofeedback constitutes a non-invasive method to observe bodily activation changes in real-time, usually by visualization on a computer screen. It is based on the concept of homeostasis, assuming the autonomous nervous system to keep activation states in balance and to steadily adapt to internal and external requirements [1]. Biofeedback applications promote our understanding of affective processing and enable voluntary interference by means of simple cognitive strategies. Clinical studies provide ample evidence for a long-term reduction of anxiety symptoms on basis of biofeedback therapy [2,3]. Thereby, patients are exposed to fear-laden stimuli and acquire self-efficacy by exercising influence on pathological reactions (for example, extremely increased heart rate activity). To enable effective control, visual feedback is usually provided directly, continuously and unaltered. However, treatment durations in biofeedback exhibit strong inter-individual variations and first successes can often be achieved only after about twenty sessions [4]. This raises the question whether the envisaged changes in affective processing can be accelerated or in some way influenced in the desired direction.

Barlow [5] presents a model of panic disorder and emphasizes that patients develop exaggerated fears of somatic symptoms associated with panic attacks. This apprehension is then again accompanied by increased autonomic arousal and vigilance towards bodily changes which results in a vicious cycle of concerns and physiological activation. After comprehensive cognitive restructuring, however, trainees are often surprised to experience the differences between physiological responses from their fearful thoughts compared to responses from their restructured thinking [6]. The current study explores a rather untapped approach to assist and accelerate this process by providing sham feedback on physiological responses. Sham feedback constitutes a barely applied control condition in which participants receive feedback that does not correspond to their bodily state. The presentation of feedback that suggests weak autonomic reactions to a negative stimulus may thereby reduce the overall physiological responsiveness by restructuring cognitive processing in terms of an attitude change towards the stimulation. Previous studies that apply false or altered signals to influence bodily reactions mainly address feedback on heart rate activity–an event which involves rather easy-to-detect inconsistencies for the individual participant. Accordingly, there is little consensus among the relevant literature. Valins [7] provides male participants with pictures of naked females and observes higher ratings of attractiveness during the presentation of sham feedback that suggests a fast heartbeat. Valins and Ray [8] reduce the fear of snakes in phobic patients by presenting manipulated feedback on heart rate activity; however, their findings could not be replicated in a later study [9]. Ehlers et al. [10] observe higher arousal ratings in patients with panic disorder during the presentation of sham feedback that suggests increased heart rate activity. An actual increase in heart rate activity could not be observed. Besides feedback properties, such inconsistencies may also be linked to the concept of interoception which describes the sensitivity for stimuli from the inside of the body [11]. According to [12], interoceptive sensibility plays a decisive role in biofeedback performance and may also moderate the individual outcome during sham feedback processing.

The present sham feedback study explores the applicability of two peripheral measures, pupil size changes (Experiment 1) and skin conductance responses (SCR, Experiment 2). Both measures have demonstrated to depict changes in bodily arousal and to allow a reliable assessment of the affective state [13,14]. Also, they have been implemented successfully in recent studies on biofeedback [15,16], whereas, to our knowledge, neither of the two have been applied in sham feedback research yet. Whether this is due to a publication bias or these measures are actually suited to support the proposed technique will be examined hereinafter. Overall aim is to explore future standards for the application of sham feedback in research and therapy.

## Experiment 1

### Aim

Sham feedback is supposed to influence cognitive assessment, which, in turn, retroacts on the actual bodily arousal. Successful manipulation with regard to weakening the reaction to (fear-driven) stimulation should therefore be reflected primarily in the physiological responsiveness. Or, more precisely, in smaller mean values compared to affective processing in a true biofeedback condition. Experiment 1 applies a fully controlled biofeedback study that generates individualised sham feedback from ongoing pupil dynamics and connects it to various negative and positive auditory stimuli.

### Materials and methods

#### Design and procedure

Experiments took place in a windowless laboratory, environmental conditions were kept constant at a room temperature of 21°C and an ambient illumination of 200 lux. Participants were seated in an upright position at a small table with their head put on a chin rest. A video-based eye-tracker was attached to a computer screen at a distance of 60 cm. The cover story suggested to investigate interoceptive perception during affective processing. Participants were requested to listen to auditory stimuli while simultaneously monitoring real-time feedback on pupil size changes and check it against their bodily arousal. Sound sequences were provided via headphones.

We applied a within-subject design. Three negative, three positive and three neutral stimuli were presented, each over a period of six seconds. Sound sequences were taken from the International Affective Digitized Sound System 2 (IADS-2) [17]. Stimuli were selected according to average valence and arousal ratings. Valences of the neutral stimuli were restricted to ratings between 4 to 6, arousal ratings were supposed to be lower or equal to 4. Based on a valence-arousal plot we selected the three sounds with the shortest distance to each other (valence: M: 5.81, SD: 1.92; arousal: M: 3.80, SD: 1.89). Arousal values for negative and positive stimuli involved ratings equal to or higher than 7. Negative stimuli were restricted to the category “human screams” with valence ratings equal to or lower than 3. Again, three stimuli with the shortest distance to each other were selected (valence: M: 1.87, SD: 1.46; arousal: M: 7.91, SD: 1.76). Positive stimuli featured a valence rating equal to or higher than 7 and comprised cheering crowds and cheerful music (valence: M: 7.50, SD: 1.67; arousal: M: 7.11, SD: 2.02).

Real-time feedback of pupil size changes was provided as a circle that behaved proportionally to actual diameter; sham feedback indicated either a strong (large diameter) or weak (small diameter) activation. Each stimulus category (negative, positive, neutral) was coupled with all three types of feedback, resulting in nine stimulus-feedback combinations and a total number of 27 trials. All conditions were randomly assigned to keep the procedure balanced between participants.

**Fig 1:**
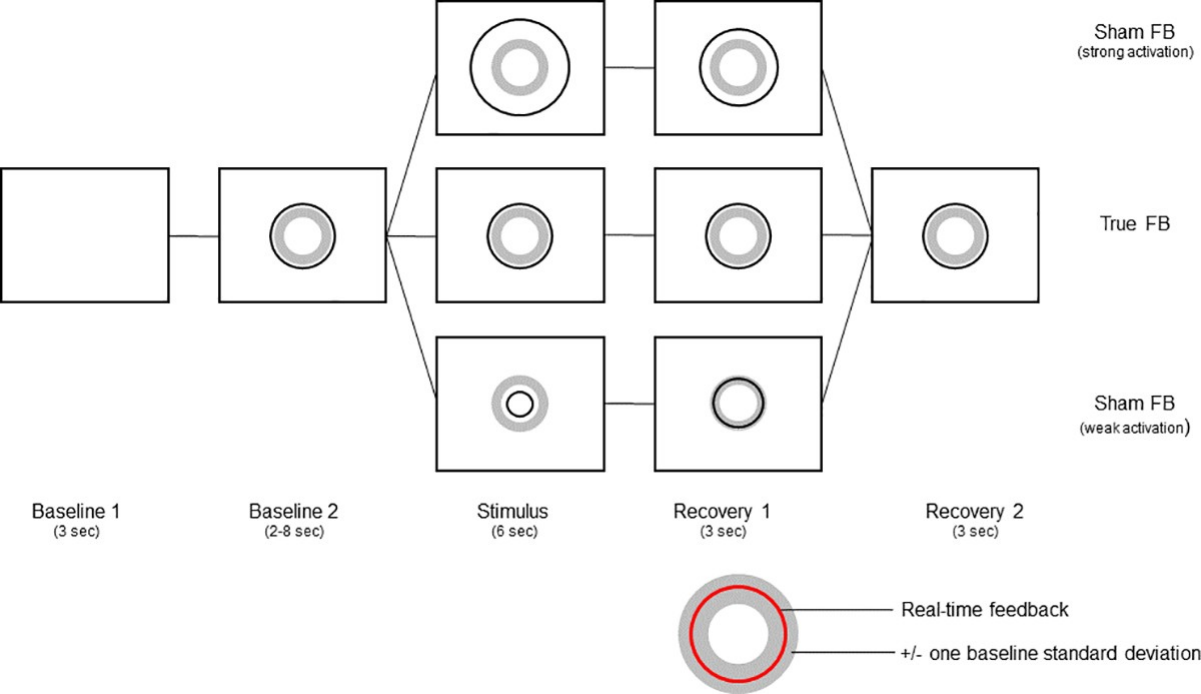
Experimental procedure. Sequences during true pupillary feedback (central row) and sham feedback trials (weak: Lower row; strong: Upper row). The dark circle (coloured red in the original display) depicts pupil size changes in real-time; the grey circle indicates one standard deviation of the average pupil diameter during baseline acquisition.

Baseline acquisition preceded every trial. Baseline 1 (3s) was applied to generate a dark grey ring that illustrates the range of spontaneous pupil size changes (one standard deviation beyond baseline mean). Baseline 2 includes real-time feedback on pupil diameter in shape of a dynamic red circle and was applied with a randomized duration of 2, 4, 6 or 8 seconds to avoid anticipation effects before stimulus-onset. Hereafter, negative, positive or neutral auditory stimulation was provided (6s). During sham feedback trials, real-time feedback on pupil diameter was distorted and the red circle either enlarged or reduced according to the formula depicted below (Real-time data filtering and feedback). During recovery period 1 (3s) this bias was steadily removed until true feedback was presented again for another three seconds (recovery period 2). The experimental procedure is depicted in Fig 1. Feedback scheme was depicted against a light grey background. Each trial was initiated by a button press. Actual pupil diameter served as the dependent variable.

In addition to the normative data provided by Bradley & Lang [17], all sound sequences were rated on a 9-point scale with regard to the valence and arousal dimension using the Self-Assessment Manikin (SAM). The SAM-Rating consists of two scales including the sketch of a manikin miming nine different states of valence and arousal. The valence scale ranges from 1 (negative) over 5 (neutral) up to 9 (positive), whereas the arousal scale involves values from 1 (relaxed, calm) to 9 (aroused, nervous).

At the end of the experiment, each volunteer filled out five subscales (noticing, attention regulation, emotional awareness, body listening and trusting) of the Multidimensional Assessment of Interoceptive Awareness (MAIA) [18] to determine the general ability for interoceptive sensibility. The concept of interoceptive awareness describes the individual’s dispositional ability to detect internal bodily sensations [19]. Internal consistencies of the selected subscales range from acceptable to good (Cronbach’s α = .69-.87), whereby all 22 items are rated on a 5-point scale from 0 (never) to 5 (always). The higher the score, the better the interoceptive sensibility. Also, participants were asked to complete the Short Suggestibility Scale (SSS) [20], to measure the extend of suggestibility, that is, the response to individual manipulation [21]. The SSS comprises 21 items that are to be rated on a 5-point Likert-scale (from “do not agree at all or only slightly” to “strongly agree”) featuring a strong internal consistency (Cronbach’s α = .86 for students). For a straightforward interpretation, a sum score was calculated featuring 21 to 105 points. Higher values indicate a strong suggestibility. The last questionnaire to be filled out was the trait-anxiety scale of the State-Trait-Anxiety Inventory [22] which assess the general disposition to react anxiously. 20 items were rated on a 4-point Likert-scale (from “almost never” to “almost always”). Again, a sum score was calculated ranging from 20 to 80 and converted into gender and age specific T-scores (M: 50, SD: 10). The higher the T-score, the higher the individual trait anxiety [22]. Finally, the participants had the opportunity to comment on conspicuous features and point out special incidents or irregularities. Afterwards, all volunteers were informed about the true objective of the experiment.

#### Real-time data filtering and feedback

Feedback scheme was designed according to the layout suggested by Ehlers et al. [23,24]. To indicate the range of task-unrelated fluctuations, one standard deviation beyond the mean of baseline 1 (3s) was depicted as a dark grey ring. Actual pupil diameter was visualized as a red circle. To reject blink artifacts, real-time data filtering was applied according to the approach validated in Ehlers et al. [23] and Georgi et al. [25]. Thereby, specifications of Bremner [26] are converted to the current sampling rate of 60 Hz, leading to an allowable sample-to-sample change of 0.09 mm. If the distance between two values exceeded this range, the latest value was substituted by the last valid sample. For optimal usability, feedback was proportionally enlarged 35 times which results in a visual angle of approximately 6°.

During the presentation of sham feedback that suggests a strong autonomic activation, pupil size changes were manipulated in accordance with the natural pupillary response to auditory affective stimulation [27]. Originating from the participant’s actual pupil diameter, sham feedback was realized on basis of logarithmically distorted, recursively defined real-time feedback indicating either a strong (sharp increase in diameter) or weak activation (decrease in diameter). During pilot testing, it became obvious that participants repeatedly indicated “weak” sham feedback to strongly differ from their bodily experience. The level of distortion was then gradually reduced until an all too clear imbalance was eliminated. That is, until participants did no longer indicate that they experience an “all too strong mismatch” between feedback dynamics and interoceptive awareness. As a consequence, sham feedback was unevenly distorted between “weak” and “strong” activations.

Manipulation was applied with stimulus-onset at time 0. The variable “time” was always initiated with a value of 1 while 1/60 was continuously added for nine seconds (6s auditory stimulation and 3s recovery period 1), indicating feedback to be updated 60 times per second. The variable “i” serves as a counter whereas “fake activation” defines the sham feedback type (either strong or weak). Sham feedback based on true pupil diameter was calculated as follows:

i = 1

Δt = $\frac{1}{60}$

sham feedback [0] = 0

time [0] = 1

feedback [i] = true diameter [i-1]–sham feedback [i-1]

time [i] = time [i-1] + Δt

if fake activation = = strong:

        sham feedback_strong_ [i] = sham feedback_strong_ [i-1]– 15 $\times \left(\frac{\log(\mathrm{time}\;[i])-\log\left(\mathrm{time}\;[i‐1]\right)}{\log\left(3\right)}\right)$

else:

        sham feedback_weak_ [i] = sham feedback_weak_ [i-1] + 1 $\times \left(\frac{\log(\mathrm{time}\;[i])-\log\left(\mathrm{time}\;[i‐1]\right)}{\log\left(3\right)}\right)$

i = i+1

With sham feedback_strong_ indicating a strong activation and sham feedback_weak_ indicating a weak activation.

To prevent an abrupt end of feedback distortion after stimulus-offset (6s), sham feedback was slowly withdrawn over a period of three seconds (recovery period 1). The steady decline was realized as follows:

feedback [i] = true diameter [i-1]–sham feedback [i-1]

time [i] = time [i-1] + Δt

if fake activation = = strong:

        sham feedback_strong_ [i] = sham feedback_strong_ [i-1] + 20 $\times \left(\frac{\log(\mathrm{time}\;[i])-\log\left(\mathrm{time}\;[i‐1]\right)}{\log\left(3\right)}\right)$

else:

        sham feedback_weak_ [i] = sham feedback_weak_ [i-1]– 35 $\times \left(\frac{\log(\mathrm{time}\;[i])-\log\left(\mathrm{time}\;[i‐1]\right)}{\log\left(2\right)}\right)$

i = i+1

#### Pupil data post-processing

To make individual pupil size changes comparable, average values of the last 1.5 seconds of baseline 2 were subtracted from each value during stimulus processing. According to Ehlers et al. [23], pupil diameter remains relatively stable over a period of 200–250 ms. Given the current sampling rate of 60 Hz, we considered 19 or more consecutive values (316.67 ms) as measuring errors and replaced them via linear interpolation during post-processing. Less than 60% of data per trial led to the exclusion of a dataset. In total, 3% of trials had to be sorted out.

#### Apparatus

Pupil diameter was measured with an iView X SMI RED Eye-Tracker (SensoMotoric Instruments) featuring a sampling rate of 60 Hz. The iView X software (v. 2.8.43), the iView X API software (v. 3.7.10) and the open source toolbox PsychoPy (v. 1.85.1) [28] were applied for real-time visualization, data recording and storage. Feedback was displayed on a BenQ G2200WT monitor featuring a WSXGA+ resolution of 1680 x 1050 pixels. Rating scales were presented with PsychoPy, questionnaires were provided via the online-survey tool LimeSurvey (v. 2.73.1) [29]. Statistical analyses were carried out with IBM SPSS Statistics (v. 24).

#### Participants

25 volunteers participated in Experiment 1. During post-test interviews, participants were provided with the opportunity to comment on their experience during biofeedback processing and to point out special incidents or irregularities. One participant indicated that feedback data seemed independent of her bodily reactions. Confidence in the validity of signals constitutes a central requirement in biofeedback and reflecting on sham feedback may distract from the underlying objective of focusing on interoceptive perception. As a consequence, we decided to exclude the participant from further analyses; 24 participants remained (19 females; mean age 25 years, SD: 10). All had normal hearing and normal or corrected-to-normal vision. They specified no history of head injury and no neurological or psychiatric disorders. Six participants reported prior experiences with biofeedback applications and it is conceivable that such knowledge alters the nature of sham feedback processing. However, these participants differ widely with regards to previous experiences; one assisted in co-supervising a biofeedback study, another reported prior experience with skin conductance biofeedback. The remaining three participants indicated knowledge on heart rate biofeedback sessions. Written informed consent was obtained before start. All measurements were performed in accordance with the Declaration of Helsinki and approved by a local ethics committee (Ethikkommission, Ulm University).

### Results

Participants obtained an average trait anxiety score [22] of 42.5 (SD: 10.9) (mean T-score: 57.1 (SD: 9.0)), indicating an average fear disposition. The level of suggestibility [20] amounted to 48.9 (SD: 9.8) (standard values for students: M: 53.8 (SD: 10.9)), suggesting a moderate suggestibility. To assess the ability for interoceptive awareness [18], we calculated values from all five subscales. Participants obtained a mean of 2.9 (SD: 0.8) on the combined scale, indicating an average ability for interoception. We carried out a correlation analysis between psychometric variables and average pupil diameter. Since the results did not differ from separate correlations between feedback types and psychometric variables, average pupil diameters across all feedback conditions were combined. However, a linear relationship could not be observed (trait-anxiety: r = .18, p = .399; suggestibility: r = -.29, p = .176; interoceptive awareness: r = -.20, p = .352).

Table 1 depicts the participants’ valence and arousal ratings across all categories of stimulation. Positive sounds were rated less arousing and neutral sounds slightly more pleasant compared to the sample in Bradley and Lang [17].

**Table 1:** Descriptive statistics of the SAM-ratings across stimulus categories.

	Valence				Arousal			
Sound category	M	SD	MIN	MAX	M	SD	MIN	MAX
Neutral	**6.21**	1.79	1.00	9.00	**3.47**	1.74	1.00	8.00
Negative	**2.43**	2.13	1.00	9.00	**7.46**	1.40	3.00	9.00
Positive	**7.00**	1.48	2.00	9.00	**5.38**	2.03	1.00	9.00

A Friedman-test indicates significant differences between positive, negative and neutral stimuli in valence and arousal ratings (valence: χ2(2) = 79.14, p < .001, W = 0.55; arousal: χ2(2) = 97.01, p < .001, W = 0.67). Bonferroni-corrected post-hoc tests were carried out using Wilcoxon-tests. For the valence dimension, all sound categories were rated significantly different from each other (neutral-negative: z = -6.21, p < .001, r = -.73; neutral-positive: z = -2.49, p = .013, r = -.29; negative-positive: z = -6.96, p < .001, r = -.82). The same was observed in arousal ratings ((neutral-negative: z = -7.19, p < .001, r = -.85; neutral-positive: z = -5.75, p < .001, r = -.68; negative-positive: z = -6.14, p < .001, r = -.72). Accordingly, negative stimuli were rated as the least pleasant and most activating sounds, whereas positive stimuli were rated as the most pleasant sounds. Neutral stimuli were assessed as least arousing.

Participants were requested to evaluate the concordance between feedback dynamics and bodily sensations after each trial on a scale from 0 (no match at all) to 100 (perfect match). A two-way repeated measures ANOVA (Greenhouse-Geisser corrected) revealed a significant interaction between stimulus category and feedback type (F(4, 88) = 5.28, p = .001, η^2^_partial_ = 0.2, ε = .78). For negative stimuli we observed considerable differences with regards to feedback category (F(2, 46) = 11.13, p < .001, η^2^_partial_ = 0.3). Post hoc testing (Bonferroni-corrected) indicated significant differences between true feedback and “strong” sham feedback (t(23) = -3.11, p = .005, d = 0.7), as well as between “strong” and “weak” sham feedback (t(23) = 4.21, p < .001, d = -1.05). Highest correspondence ratings could be observed during trials that combined negative stimuli with sham feedback that suggests strong activation. In contrast, only little correspondence was indicated during the combination of negative stimuli with sham feedback that suggested weak activation. For positive stimuli, we did not observe any influence of feedback on the rating. Descriptive statistics on concordance ratings are depicted in Table 2.

**Table 2:** Concordance ratings.

	Concordance rating	
Sound category x feedback type	M	SD
**Neutral**		
True biofeedback	64.46	13.84
Strong sham feedback	64.03	17.77
Weak sham feedback	65.73	12.29
**Negative**		
True biofeedback	65.88	14.19
Strong sham feedback	76.25	13.45
Weak sham feedback	61.33	14.99
**Positive**		
True biofeedback	66.83	12.03
Strong sham feedback	64.06	16.48
Weak sham feedback	66.88	15.00

We applied a correlation analysis to determine the connection between stimulus-evoked pupil size changes and stimulus-based arousal ratings across all feedback conditions. During true feedback trials, average pupil diameter tends to be positively correlated with the average arousal valuation (r = .37, p = .075). Providing sham feedback that indicates a weak reaction strengthens this connection (r = .50, p = .013). Pupil diameter during sham feedback that suggests strong responses did not reveal any connection with stimulus-based arousal ratings.

**Fig 2:**
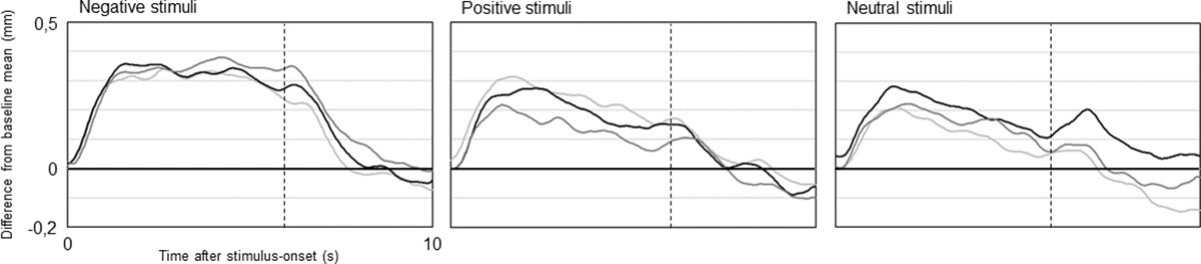
Total averages of pupil size changes during experimental conditions. Total averages (n = 24) of pupillary reactions to auditory stimuli from negative, positive and neutral categories while providing true feedback (light grey) and sham feedback suggesting strong (black) and weak activation (dark grey). Signal courses depict differences from baseline mean (set to zero on the y-axis). Vertical dotted lines indicate end of stimulation (after six seconds). For purposes of presentation, curve progressions are smoothed using a sliding average of half a second (30 data points).

With regard to the impact of stimulus category and feedback type on pupil diameter, a two-way repeated measures ANOVA (Greenhouse-Geisser corrected) revealed a significant effect of stimulus category (F(1.57, 36.06) = 9.02, p = .001, η^2^_partial_ = 0.3, ε = .78) but no influence of feedback modality (Fig 2). Post hoc comparisons indicated negative sequences (M: 0.21, SD: 0.19) to provoke stronger dilations compared to neutral (M: 0.10, SD: 0.17; mean difference: 0.11 mm; p< .001, d: 0.6) and positive stimuli (M: 0.12, SD: 0.19; mean difference: 0.09 mm; p = .002 d: 0.5), whereas pupillary reactions to neutral and positive sounds did not differ. Also, pupil dilations evoked by negative sequences continue to remain about five seconds; in contrast, dilations from positive and neutral stimuli peak briefly and immediately fall back on baseline level.

Descriptive statistics of baseline-corrected pupil size data are depicted in Table 3.

**Table 3:** Baseline-corrected pupil size data divided by category and feedback type.

	Pupil diameter (baseline-corrected)	
Sound category x feedback type	M	SD
**Neutral**		
True biofeedback	0.05	0.15
Strong sham feedback	0.15	0.17
Weak sham feedback	0.09	0.17
**Negative**		
True biofeedback	0.19	0.18
Strong sham feedback	0.21	0.14
Weak sham feedback	0.23	0.24
**Positive**		
True biofeedback	0.15	0.19
Strong sham feedback	0.16	0.15
Weak sham feedback	0.05	0.22

**Fig 3:**
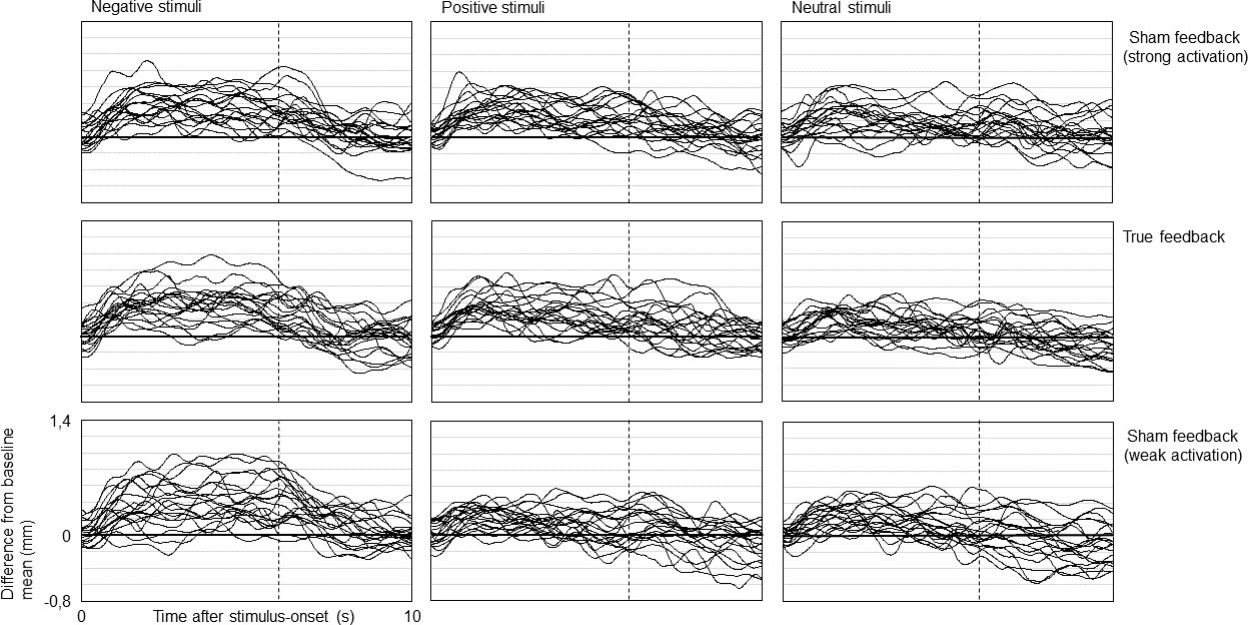
Individual averages of pupil size changes during experimental conditions. Individual averages (n = 24) of pupillary reactions to auditory stimuli from negative, positive and neutral categories while providing true feedback (middle row) and sham feedback suggesting strong and weak activation (upper/lower row). Signal courses depict differences from baseline mean (set to zero on the y-axis). Vertical dotted lines indicate end of stimulation (after six seconds). For purposes of presentation, curve progressions are smoothed using a sliding average of half a second (30 data points).

To indicate interindividual differences within stimulus-feedback combinations, Fig 3 depicts individual averages of all participants across all conditions.

### Discussion

Stimulus category (negative, positive, neutral) exerts a considerable influence on pupil diameter. In accordance with higher arousal ratings on the SAM scale (Table 1), negative auditory stimuli induce stronger pupil dilations compared to positive and neutral sequences. This is in line with observations from Bradley et al. [13] and Al-Omar et al. [14] who report negative stimuli or feelings of anxiety to induce the largest pupil diameters among all basic emotions. However, it contradicts findings from Janisse [30] as well as from Partala and Surakka [27] who observe pupil diameter to behave curvilinear on the valence scale with strongest dilations at negative and positive ends. As indicated above, sound sequences were selected carefully and in accordance to standardized ratings. However, participants still rated them slightly different from the normative sample [17]. This was especially true for arousal ratings concerning positive stimuli which were assessed less activating in the current sample. Also, mean arousal ratings for positive stimuli were lower than for negative sequences. Partala and Surakka [27] observe similar deviations from normative ratings but in the opposite direction. They report negative and positive sound sequences to be rated more arousing as well as more negative or pleasant and conclude that arousal constitutes the decisive influencing factor on pupil diameter. This relationship might be a reason for smaller pupil diameters while processing positive compared to negative content. The normative sample does not provide further positive stimuli featuring arousal ratings above seven; to assure preferably strong affective reactions, we focused on negative stimulus processing during Experiment 2.

The correlation analysis indicates a tendency towards a linear relationship between stimulus-based arousal ratings and pupil diameter. Bradley and Lang [31] observe a similar correlation for amplitudes of SCR and the assessment of acoustic stimuli. In the current work, this trend increases during the presentation of sham feedback that suggest weak activation. How can this be explained? While evaluating the match between feedback behaviour and interoceptive awareness, participants experience especially high correspondence during trials that combine negative stimuli with sham feedback that suggests strong activation. It can therefore be stated that amplifying the natural response (dilation) during stimulus processing that is associated with high arousal ratings (negative sounds) appears plausible to naïve observers. In contrast, a reduction of pupil diameter after stimulus onset (which does not occur during stimulation of any kind) is evaluated as not convincing, probably irritating. Such confusion may build upon the stimulus-induced effect by further enlarging pupil diameter and strengthen the correlation between arousal rating and pupil size. We therefore assume that the absence of a reaction (or only a comparatively small response) constitutes a more sensible approach to suggest low activation and to reduce physiological responsiveness via sham feedback.

As can be learned from the presentation of individual signal characteristics (Fig 3), pupil diameter exhibits a high degree of fluctuation which is reflected in the ongoing feedback dynamics. In case of pupils that feature a rather poor responsivity it must be assumed that manipulation in either direction may not always be fully recognized. It is therefore possible that individual participants were hardly aware of any incorporated changes in feedback behaviour.

Taken together, individualised sham feedback from ongoing pupil dynamics does not influence affective processing in the desired way. However, the current results provide valuable indications as to the way of designing sham feedback responses. Experiment 2 applies manipulated responses to negative stimuli based on intuitive, pre-recorded SCR.

## Experiment 2

### Aim

Experiment 2 builds upon our observations from Experiment 1. To ensure clear and comprehensive feedback, we apply SCR as an easy to interpret measure that is characterized by slow changes and therefore particular suited for purposeful manipulation. To control for overly strong interindividual variations, we provide pre-recorded signal courses that are exclusively inspired by natural responses. Also, we focus on negative stimulation since positive processing could not be distinguished from responses to neutral stimuli and was not affected by sham feedback in any way. Standardised inventories like the IADS comprise a variety of negative stimulus sequences that include both complex acoustic scenarios as well as simple noises. To cover a preferably wide range, we provide sequences from four distinct categories.

### Materials and methods

#### Design and procedure

Experiments took place in a windowless laboratory, environmental conditions were kept constant at a room temperature of 21°C and an ambient illumination of 200 lux. Participants were seated in an upright and relaxed position at a small table. Right hand was put onto a soft bolster and skin conductance sensors were attached to tips of index and middle finger. Participants were suggested to be part of the control group in a biofeedback study and instructed to passively listen to neutral and negative sound sequences while simultaneously monitoring real-time feedback of SCR on a computer screen. Sound sequences were provided via headphones. Participants were requested to avoid any movement during data acquisition.

We applied a within-subject design. Three neutral and 12 negative auditory stimuli were presented, each over a period of six seconds. All sequences were taken from the IADS-2 [17]. Negative sounds were obtained with comparable valence and arousal ratings from four categories (three sounds per category): “Scream” (being the same as during Experiment 1), “fight”, “violence” and “accident” (negative stimuli: valence: M: 1.86, SD: 0.22; arousal: M: 7.69, SD: 0.30; neutral stimuli: valence: M: 5.10, SD: 0.21; arousal: M. 3.73, SD: 0.73). Feedback on SCR was provided as time course in a two-dimensional coordinate system. Feedback type was presented block-wise with true feedback during Block 1 and sham feedback during Block 2. Actual SCR served as the dependent variable.

**Fig 4:**
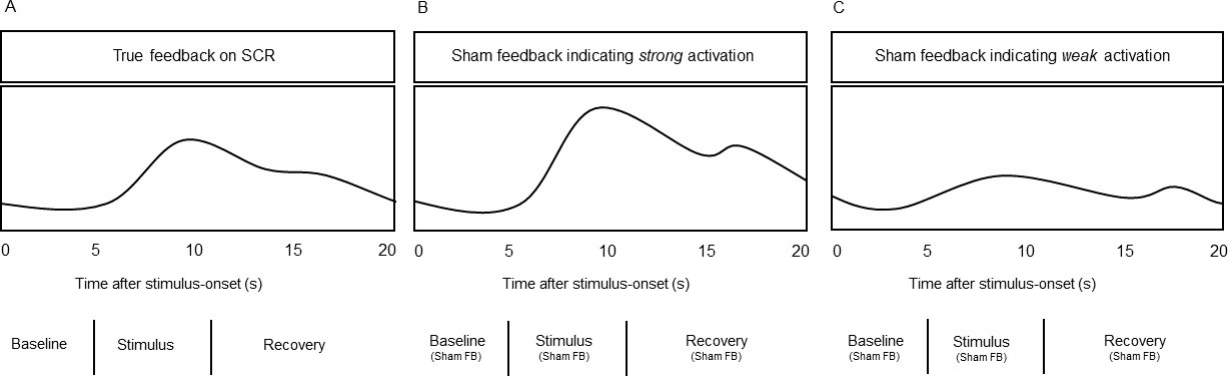
Experimental procedure. Exemplary signal characteristics during Block 1 (true feedback, A) and Block 2 (strong (B) and weak (C) sham feedback).

During Block 1, we provided three neutral and four negative stimuli, the latter taken in equal parts from all four aversive categories. True feedback on SCR was continuously fed back across an entire trial (Fig 4A). The first five seconds served as a baseline; hereafter, auditory stimulation was applied (6s). Feedback then remained on the screen for another nine seconds (recovery period). The next trial started after a five second pause. Axis labels that inform about the magnitudes of changes were not depicted. The sequence of neutral and negative stimuli was hard-coded and involved a fixed order without two of the same (negative) category being consecutive.

After a one-minute break, Block 2 measuring was carried out. To compensate for habituation effects, negative sequences were replaced by eight new stimuli, two out of each aversive category. Neutral sounds were no longer provided. Instead of true biofeedback we applied eight pre-programmed signal courses, indicating either strong (4x) or weak (4x) SCR. To suggest strong responses (Fig 4B), sham signals were modelled according to natural but pronounced SCR to negative stimuli. That is, signals exhibit a sharp increase one to two seconds after stimulus-onset, attain a maximum after four to five seconds and then slowly fall back to pre-stimulus level. To suggest the lack of a considerable SCR (Fig 4C), sham signals exhibit only a small response after stimulus-onset. Each participant was exposed to two negative stimuli of each category, one associated with a “strong”, the other one with a “weak” sham response. As during Block 1, stimulus presentation followed a predetermined order without two of the same negative category being consecutive.

During Experiment 1, we did not encounter differences between individual (arousal) ratings of negative stimuli and the normative data provided by Bradley & Lang [17]. We therefore refrained from individual assessments at this point. However, participants were asked to fill out a questionnaire including the opportunity to comment on conspicuous features during task processing. Finally, every participant was informed about the true objective of the experiment.

#### SCR data post-processing

To make individual results comparable, averages of the five-second baseline were subtracted from each value during stimulus processing and recovery period. For statistical analyses, we calculated average values of SCR across the complete 15 seconds after stimulus-onset. This period includes the time after stimulus presentation which is in accordance with Venables and Fletcher [32] as well as Levander et al. [33] who emphasize the functional role of the recovery time as an independent variable.

#### Apparatus

SCR were measured with Ag/AgCL electrodes of a NeXus-10 Mark II (Mind Media) featuring a sampling rate of 32 Hz. BioTrace+ software was applied for real-time feedback, sham feedback visualization, data recording and data storage. Feedback was displayed on a BenQ G2200WT monitor featuring a WSXGA+ resolution of 1680 x 1050 pixels. Statistical analyses were carried out with the open source software JASP (v. 0.10) [34].

#### Participants

28 volunteers participated in the present study. In post-test interviews, four of them indicated that feedback during Block 2 did not consistently match their bodily experience. Sham feedback is supposed to influence cognitive assessment and thus bodily arousal. It is reasonable to assume that scepticism or uncertainty about the validity of feedback prevents any proper interference. Also, it cannot be ruled out that these participants identified the true objective of the study already during task processing. As during Experiment 1, the data sets were removed from further analyses. 24 participants remained (21 females; mean age 25 years, SD: 8), all had normal hearing as well as normal or corrected-to-normal vision. They specified no history of head injury and no neurological or psychiatric disorders. Two of them reported prior experiences with SCR-based biofeedback applications. Written informed consent was obtained before start. All measurements were performed in accordance with the Declaration of Helsinki and approved by a local ethics committee (Ethikkommission, Ulm University).

### Results

Baseline activity featured a high inter-individual variability: during Block 1, individual averages ranged from 1.30 to 15.67 μS (M: 5.75, SD: 3.95); during Block 2 we observed a similar distribution (range: 1.18 to 21.62 μS; M: 6.62, SD: 5.13). Statistical differences in baseline activity between both blocks did not occur.

The assumption check of normality indicated that our data are not well modelled by a normal distribution. We applied an outlier analysis (individual averages 1.5 times the interquartile range above/below the upper/lower quartile are rejected) on SCR to neutral and negative stimuli (averaged across all categories) during Block 1 and removed another four participants. Accordingly, the following results are based on a data pool of 20 participants.

**Fig 5:**
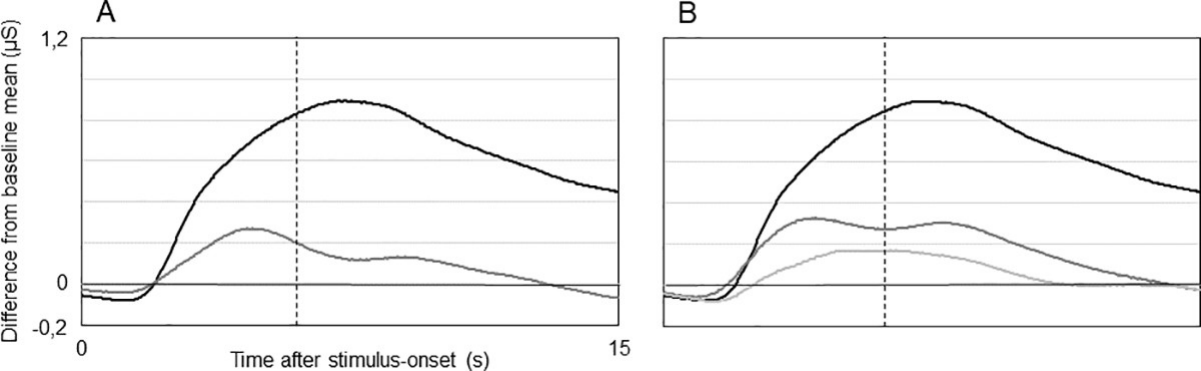
Averaged SCR to auditory stimuli during true biofeedback and sham feedback. A: Total averages (n = 20) of SCR to negative (black) and neutral (grey) auditory stimuli while providing true biofeedback. B: Total averages (n = 20) of SCR to negative auditory stimuli while providing true biofeedback (black) and sham feedback suggesting strong (dark grey) and weak activation (light grey). Signal courses depict differences from baseline mean (set to zero on the y-axis). Vertical dotted lines indicate end of auditory stimulation (after 6s). For purposes of presentation, curve progressions are smoothed using a sliding average of one second (32 data points).

The analyses within Block 1 served as a manipulation check. We conducted a paired sample t-test to determine differences in SCR to neutral and negative stimuli (averaged across all categories) while providing true biofeedback. Results support findings from Bradley et al. [13], indicating considerably stronger reactions to negative (M: 0.31 μS, SD: 0.36) compared to neutral stimuli (M: 0.02 μS, SD: 0.15; t(19) = -3.856, p< .001, d: 0.8). Fig 5A depicts the averaged signal courses; values indicate differences from baseline mean. Negative sound sequences are accompanied by a sharp increase in SCR approximately one to two seconds after stimulus-onset. The signal peaks shortly after stimulus-offset and then slowly decreases towards baseline level.

SCR to negative sound sequences turned out to be affected by stimulus category (“scream”, “fight”, “violence”, “accident”). During true biofeedback, a one-way repeated measures ANOVA revealed considerable differences between the means (F(2.569, 48.8) = 9.835, p< .001, η^2^_partial_ = 0.3, ε = .64). We report a corrected F-statistic (Greenhouse-Geisser), post hoc testing was carried out using the Bonferroni correction. Fight and scream stimuli provoked the strongest SCR and did not differ from each other. Fight sounds induced stronger reactions compared to neutral sequences (p< .001, d: 1.3) but also compared to accident (p = .043, d: 0.7) and violence stimuli (p = .025, d: 0.8). Scream sounds induced stronger SCR compared to neutral (p = .049, d: 0.7) and accident stimuli (p = .030, d: 0.7). SCR to accident and violence sounds did not differ and could not be distinguished from reactions to neutral stimuli.

Besides stimulus category, feedback type (true biofeedback vs. sham feedback) modulated affective processing. A one-way repeated measures ANOVA (Greenhouse-Geisser corrected) revealed significant differences between SCR to negative stimuli (averaged across all categories) while observing true biofeedback and sham feedback suggesting strong or weak reactions (F(1.392, 26.5) = 13.364, p<. 001, η^2^_partial_ = 0.4, ε = .70) (Fig 5B). Post hoc comparisons indicated stronger SCR during true biofeedback trials compared to sham feedback suggesting strong (M: 0.08 μS, SD: 0.29; mean difference: 0.23 μS; p = .021, d: 0.7) and weak responses (M: -0.003 μS, SD: 0.22; mean difference: 0.32 μS; p< .001, d: 1.0). Sham feedback indicating strong SCR tended to induce stronger reactions compared to sham feedback suggesting weak responses (mean difference: 0.09 μS; p = .10).

To determine interactions between negative stimulus categories and feedback type, we conducted a two-way repeated measures ANOVA (Greenhouse-Geisser corrected) and observed a significant effect (F(3.093, 58.7) = 3.437, p = .021, η^2^_partial_ = 0.2, ε = .51). As reported above, violence and accident sounds provoked weak SCR that did not differ from reactions to neutral stimuli. Post hoc comparisons indicated that combining these stimuli with sham feedback (both “strong” and “weak”) did not alter affective processing. The opposite was true for fight and scream sounds: Scream stimuli connected to “weak” sham feedback induced considerably lower SCR compared to scream sequences while observing true feedback (p = .005, d: 0.9). The same accounted for scream stimuli associated with “weak” sham feedback compared to fight stimuli based on true feedback (p< .001, d: 1.2). Sham feedback (both “strong” and “weak”) that accompanies fight stimuli considerably reduced SCR compared to the presentation of true feedback (p< .001, d: 1.1). The same was true for comparisons to scream stimuli connected with true feedback (“strong”: p = .004, d: 0.9; “weak”: p = .016, d: 0.8). Descriptive statistics of baseline-corrected SCR are depicted in Table 4.

**Table 4:** Baseline-corrected SCR divided by category and feedback type.

	SCR (baseline-corrected)	
Sound category	M	SD
**True biofeedback**		
Neutral	0.02	0.15
Scream	0.40	0.54
Fight	0.52	0.42
Violence	0.17	0.36
Accident	0.16	0.38
**Strong sham feedback**		
Scream	0.18	0.47
Fight	-0.04	0.54
Violence	0,06	0.30
Accident	0.16	0.37
**Weak sham feedback**		
Scream	-0.03	0.24
Fight	0.01	0.30
Violence	-0.01	0.41
Accident	0.02	0.35

**Fig 6:**
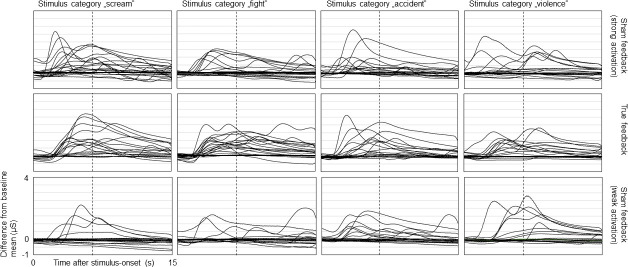
Individual SCR to auditory stimuli during true biofeedback and sham feedback. Individual SCR (n = 20) to auditory stimuli from four negative categories while providing true biofeedback (middle row) and sham feedback that suggest strong (upper row) and weak activation (lower row). Signal courses depict differences from baseline mean (set to zero on the y-axis). Vertical dotted lines indicate end of auditory stimulation (after 6s). For purposes of presentation, curve progressions are smoothed using a sliding average of one second (32 data points).

To indicate interindividual differences within stimulus-feedback combinations, Fig 6 depicts signal courses of all participants within all experimental conditions. The influence of sham feedback on affective processing is particularly visible while providing “weak” SCR during the presentation of scream and fight stimuli.

## Discussion

We applied a wide range of negative stimuli that are controlled with regards to valence and arousal ratings; however, sound sequences across categories induce different responses. Although assessed as highly arousing, stimuli from the accident and violence categories provoke SCR that cannot be distinguished from reactions to neutral stimuli. This contradicts Bradley and Lang [31] who report amplitudes of SCR to linearly increase as subjective ratings of arousal increase. In contrast, Khalfa et al. [35] observe fearful music excerpts that elicit strong SCR but are associated with low values during a subsequent intensity rating. It can therefore be noted that higher order cognitions (here: evaluative thinking) do not necessarily correlate with basic autonomous processing. Reasons for such a discrepancy are a matter of speculation. Sequences from accident and violence categories involve complex scenarios, including braking sounds, shattering glass as well as moaning or undefined noise of fighting. They occasionally appear outdated and unintentionally amusing but the context can be considered clearly negative and is probably rated accordingly. In contrast, fight and scream stimuli involve considerable SCR. Particularly scream sequences consist of terrifying sounds that forego any contextual embedding. It is conceivable that the strong physiological reaction is biologically adaptive in sense of a preparedness to react to such fear-relevant stimuli [36,37].

In contrast to Experiment 1, we present pre-recorded sham signals that unambiguously display either the absence of a SCR or a particularly strong response. As could be demonstrated, sham feedback that suggests weak SCR is suitable to reduce physiological reactions to certain stimuli, typically those that provoke strong responses. This is especially true for scream sounds. During fight sequences, however, both “weak” and “strong” sham feedback involve a considerable reduction of SCR. The reason for this finding is not entirely obvious. It is probably necessary to strictly adjust sham signals to the associated stimulus features. In case of fight sounds, this means a rapid succession of different noises. However, sham signals that suggest weak reactions are defined by simple curve progressions (slight increase, slow decline) that fit well with the dynamics of scream sequences but may prove to be inadequate or implausible during more complex stimulation. Tallying with this, four participants were able to identify the discrepancy between sham feedback and bodily experience. Further optimisation of standard signals by adapting the source of stimulation may help to avoid unambiguous results and deceive a higher number of participants.

## Conclusion

The combination of highly activating stimulus material with sham feedback that suggests weak but context-sensitive SCR enables to considerably reduce the actual bodily response within a single session. In contrast to previous studies that primarily focus the increase of autonomous arousal [7,10], the current findings indicate for the first time that biofeedback applications can be complemented by a sensible use of sham responses to support cognitive restructuring and reduce exaggerated reactions to fear-driven stimuli. However, it seems of particular importance to forego the presentation of all too unnatural reactions. Furthermore, simplicity and traceability of sham signals constitute key factors to provide convincing feedback, whereas the ambiguous nature of feedback from ongoing pupil dynamics fails to influence cognitive assessment and thus bodily arousal. Further studies on pupil-based biofeedback that feature reduced dynamics (real-time filtering) and strictly natural sham feedback may provide more promising results. For clinical application issues, however, pre-recorded sham signals should be more usable and easier to implement. Be that as it may, long-term investigations are necessary to determine whether a reduced physiological responsiveness proves to be stable over time and is also sustainably reflected in cognitive assessments.

## Supporting information

S1 Appendix (DOCX)
