# Supplementary material for: The influence of sham feedback on physiological processing during fear-driven stimulation
Source: PLoS One. 2021 May 5;16(5):e0251211. doi: 10.1371/journal.pone.0251211 (PMC8099069; doi:10.1371/journal.pone.0251211)
Supplement: S1 Appendix — (DOCX) [file pone.0251211.s001.docx]

**S1 Appendix**

In the following, numbers of the IADS-2 stimuli [17] are separately listed for each experiment.

**Experiment 1**

Neutral: 171 (CountryNight), 377 (Rain1), 602 (Thunderstorm)

Negative: 275 (Scream), 276 (FemScream2), 277 (FemScream3)

Positive: 311 (Crowd2), 352 (SportsCrowd), 817 (Bongos)

**Experiment 2**

Human scream: 275 (Scream), 276 (FemScream2), 292 (MaleScream)

Fight: 279 (Attack1), 285 (Attack2), 290 (Fight1)

Violence: 278 (ChildAbuse), 286 (Victim), 277 (FemScream3)

Accident: 422 (TireSkids), 424 (CarWreck), 600 (BikeWreck),

Neutral: 262 (Yawn), 698 (Rain2), 720 (BrushTeeth)
